# The Multimedia Piers-Harris Children's Self-Concept Scale 2: Its Psychometric Properties, Equivalence with the Paper-and-Pencil Version, and Respondent Preferences

**DOI:** 10.1371/journal.pone.0135386

**Published:** 2015-08-07

**Authors:** Mon-hsin Wang Flahive, Ying-Chih Chuang, Chien-Mo Li

**Affiliations:** 1 Department of Counseling Psychology, Chinese Culture University, Taipei, Taiwan; 2 School of Public Health, Taipei Medical University, Taipei, Taiwan; 3 Department of Electric Engineering, National Taiwan University, Taipei, Taiwan; Philipps University Marburg, GERMANY

## Abstract

A multimedia version of Piers-Harris Children's Self-Concept Scale 2 (Piers-Harris 2) was created with audio and cartoon animation to facilitate the measurement of self-concept among younger children. This study aimed to assess the psychometric qualities of the computer version of Piers-Harris 2 scores, examine its score equivalence with the paper-and-pencil version, and survey the respondent preference of the two versions. Two hundred and forty eight Taiwanese students from the first to fourth grade were recruited. In regard to the psychometric properties, high internal consistency (α = .91) was found for the total score of multimedia Piers-Harris 2. High interscale correlations (.77 to .83) of the multimedia Piers-Harris 2 scores and the results of confirmatory factor analysis suggested the multimedia Piers-Harris 2 contained good structural characteristics. The scores of the multimedia Piers-Harris 2 also had significant correlations with the scores of the Elementary School Children’s Self Concept Scale. The equality of convergence and criterion-related validities of Piers-Harris 2 scores for the multimedia and paper-and-pencil versions and the results of ICCs between the scores of the multimedia and paper-and-pencil Piers-Harris 2 suggested their high level of equivalence. Participants showed more positive attitudes towards the multimedia version.

## Introduction

Measuring the self-concept of children is strongly related to how the self-concept is defined, which theoretical model is adopted, and the influences of children’s normative development and cultural differences. Self-concept is a psychological construct of how people perceive themselves and is “essentially phenomenological in nature” [1]; therefore, it heavily depends on the self-report of children. In terms of its structure, earlier scholars viewed self-concept as a uni-dimensional organization [2–3]. Theoretically, Shavelson, Hubner, and Stanton [4] proposed a multifaceted and hierarchical model hypothesizing that the structure of self-concept has various dimensions and different levels. For example, they suggested that there are three dimensions of self-concept such as Non-academic, Academic English, and Academic Mathematics, and these dimensions can be incorporated into a higher-order general self-concept. Although scholars have different ideas about how to define these dimensions, their model has been widely accepted [5–7] and adopted by academics while designing their own self-concept measurements [1, 8–11].

The second notion is related to the descriptive and evaluative dimensions of self-concept [6, 12]. It is hard to draw a clear line between self-descriptions and self-evaluations [5]; however, Harter [6] postulated that the distinction of self-descriptions and self-evaluations may reflect the construction of self-concept and may be a result of the use of different methodologies measuring self-concept. She pointed out that people tend to conceptualize themselves and others in an evaluative perspective even at a very young age, and many instruments measuring self-concept require the participants to view self favorably or unfavorably. Some researchers focused on one perspective such as self-evaluation [3, 12]; however, many measurements of children’s self-concept include both facets [9–10, 13].

Thirdly, the normative development of children needs to be taken into account in measuring their self-concept. Children have the ability of describing and evaluating themselves from very young age [14]. From the developmental point of view, self-concept becomes more abstract and integrated with age. Harter [6] suggested that children at a very young age, such as 3- to 4-years-old, typically describe themselves in concrete and observable terms and lack cohesive self-representations. With cognitive and language development, children aged 5 to 7 can elaborate taxonomic attributes and competencies and link the opposites. Children in middle to late childhood (ages 8 to 11) can label their abilities and interpersonal characteristics, have comparative assessment with peers, and integrate opposing attributes. Therefore, to measure the self-concept of children younger than 8, the researcher needs to focus on personal attributes and competencies instead of comparative assessment with peers.

The traditional way of measuring the self-concept of children heavily relies on “verbal techniques” [5], and verbal self-description and the use of adjectives would be difficult for children to accomplish [15]. The limited cognitive or reading ability of young children may affect the application of self-concept measures. According to Piaget [16], children from six-to-seven to eleven-to-twelve are in the concrete operational thought stage of cognitive development. Children at this stage tend to use concrete materials to facilitate their abstract cognitive operations. Pure verbal or written descriptions of abstract concepts may be difficult to comprehend for young children. This could be a reason why most of the self-concept scales are designed for children older than eight years old [5]. However, incorporating audio and visual aids may assist people with difficulties comprehending these concepts [17]. Applying multimedia versions of self-report instruments would assist younger children to enhance their understanding of the instrument by linking the animation with the text and by coding the information through multiple channels including oral, pictorial, and written text presentation modes [17–19]. Older children with reading problems and lower cognitive ability may also have difficulties completing self-report scales, including self-concept scales [20–21]. In addition, young children usually have a short attention span, and studies have shown that incorporating a computer in the testing process would enhance children’s interest and enjoyment [22].

Finally, the influences of cultural differences need to be considered [6, 23–24]. Some studies found that people differ in their self construals in Western and Eastern societies [23–26]; however, others have suggested that people may differ in dimensions of self-concept because of their cultural backgrounds; nevertheless, a general pattern in self-concept can be observed [27–29]. In addition, the response style of a self-concept measure may affect its reliability and validity due to the cultural differences. Harter [6] proposed that the Self Perception Profile for Children developed by Harter [10] is not applicable to Asian children because of its response style. The items in the Self Perception Profile for Children constructed statements like “Some kids” versus “Other kids” implying the demands of social comparison. Chinese children are expected to be humble and may not be willing to show their superiority and reveal their real self-perceptions in responses. This difference affected some scales’ applicability in Asia.

In short, in measuring self-concept of young children in a non-western culture, the researcher needs to consider how the self-concept is viewed and defined and the cultural factors involved. The influences of normative development in developing self-concept of younger children need to be taken into account when designing the measurements, and adopting a multifaceted and hierarchical theoretical model is also encouraged.

The Piers-Harris Children's Self-Concept Scale 2 (Piers-Harris 2) was chosen for this study because of its multifaceted and hierarchical characteristics as well as descriptive and evaluative dimensions. It could be applicable to children as young as 7 [1, 30], and its design seems to match younger children’s cognitive and developmental abilities. The psychometric properties of the translated Chinese paper-and-pencil Piers-Harris 2 were examined, and the results suggested its applicability to Chinese children from age 6 to 15 [31–32]. Considering the developmental needs of younger children, they may benefit from the development of computer-assisted animated self-concept measures with audio or graphic features which could potentially enhance their understanding of abstract concepts such as verbal or written descriptions of self-concept.

Researchers have developed a computer program with audio recordings and cartoon animation matching the items of the Piers-Harris 2. For example, for the item “I am a happy person,” children will hear the recording through earphones while they see on the computer screen an animated picture showing a happy face with the written statement “I am a happy person” under the picture. The revision and accuracy of matching the statement to an animated picture was determined by the reviews and suggestions from two professors with a child psychology background and 8 children from 1^st^ to 4^th^ grade.

The guidelines developed by the American Psychological Association (APA) [33] stresses that the test developer should provide reliability and validity evidence for the scores of a computer-based test using the same methods as for paper-and-pencil testing. The Standards for Educational and Psychological Testing [34] also requires the developer to provide psychometric and equivalence evidence regarding different formats of psychological or educational tests. Therefore, there is a need to assess its equivalence to the scores of the paper-and-pencil version. The purpose of this research is to examine the reliability and validity evidence of the multimedia Piers-Harris Children's Self-Concept Scale 2 (multimedia Pier-Harris 2) scores, assess its equivalence with the paper-and-pencil version of Piers-Harris Children's Self-Concept Scale 2 scores (Pier-Harris 2)[13], and survey respondent preference of the multimedia Pier-Harris 2 compared to the paper-and-pencil version among Taiwanese children. It is also an attempt to extend the applications of the self-report psychological measure to younger children incorporating a multimedia format in the testing process.

## Methods

### Participants and Test Administration

Participants were 248 children (M = 131, F = 117) from the first to the fourth grade recruited from three elementary schools in the northern part of Taiwan. The size of school and the students’ socioeconomic status were considered in the process of selecting the school for the purpose of better representing the elementary schools in the northern part of Taiwan. Four hundred students were invited to participate in this study, and 248 (62%) parents gave their consent. There were 63 (25.40%) and 57 (22.98%) participants in the first and second grade; 67 (27.02%) and 61 (24.60%) participants in the third grade and fourth grade, respectively. Originally, the Piers-Harris 2 is created for children from the second grade to the twelfth grade; however, this present research attempts to further examine the applicability of the multimedia format for younger children such as first graders.

Data were collected during a four week interval. The multimedia Piers-Harris 2 was administered in the school computer lab by two trained research assistants with academic backgrounds in psychology. One research assistant taught the participants how to use the mouse to input their personal data such as grade, class as well as gender and how to answer the items on the computer screen. The other research assistant checked if any student had difficulties operating the computer and answered questions individually. Very few children had difficulties answering with a mouse and the average completion time was about 10–15 minutes.

For examining the equivalence between the scores of the multimedia and paper-and-pencil versions, participants were randomly divided into two groups. In the first testing session, group one was administered a multimedia version of Piers-Harris 2, and group two took a paper-and-pencil version. Four weeks later, group one switched to take the paper-and-pencil version, and group two took the computer one. The paper-and-pencil version was administered in the children’s regular classroom.

A questionnaire was created to investigate children’s preferences about taking the multimedia and the paper-and-pencil versions of Piers-Harris 2. The questionnaires were given to the children right after they took both versions of Piers-Harris 2.

### Ethics Statement

All ethical guidelines were followed as required for conducting human research, and written informed consents were obtained from the participants’ legal guardians. All of the participants attended this study voluntarily. The legal authorization of the translation and adaptation of Piers-Harris 2 for this research was obtained from its publishing company. The ethics committees of the Chinese Culture University approved this study and the consent procedure.

### Instruments

#### Paper-and-pencil Piers-Harris 2

The paper-and-pencil Piers-Harris 2 is a self-report test measuring children’s self-concept. It has 60 items, and each item requires a “yes” or “no” answer and denotes one of the six domains: Behavioral Adjustment (BEH), Intellectual and School Status (INT), Physical Appearance and Attributes (PHY), Freedom from Anxiety (FRE), Popularity (POP), Happiness and Satisfaction (HAP). Piers and Harris [35] reported reliability coefficients for 3^rd^, 6^th^, and 10^th^ graders as .72, .71, and .72, respectively for the original Piers-Harris Children’s Self-Concept Scale scores. Internal consistency estimates of paper-and-pencil Piers-Harris 2 scores for the total score was .91, and for the six domains they ranged from .74 to .81. Various pieces of validity evidence were provided for the scores of the paper-and-pencil Piers-Harris 2 by Piers and Herzberg [1]. The total score and the scores of the six domain scales showed strong interscale correlations ranging from .84 to .73. An exploratory factor analysis was conducted and yielded six factors supporting its multidimensional traits. Low to moderate correlations were found with the scores of the Aggression Questionnaire [36], the Attitudes Toward Guns and Violence Questionnaire [37], the Overeating Questionnaire [38], and My Worst Experience Scale [39].

#### Multimedia Piers-Harris 2

Piers-Harris 2 was translated into Chinese using forward translation by two independent translators, one with a doctoral degree and the other with a master degree in child psychology. Forward translation is used because self-concept is concept-mediated oriented emphasizing the connection of ideas instead of word association [40–41]. A software program using Macromedia Flash MX and C++ computer languages was developed for the multimedia Pier-Harris 2. Each item was displayed on the computer screen with a statement and an animated cartoon matching the description of the statement while a voice recording of the statement was played at the same time. A pair of headphones was used to listen to the voice recording, and a computer mouse was used to click the answer on the computer screen under the cartoon animation.

#### The Elementary School Children’s Self Concept Scale (ESCS)

The ESCS measures children’s self-concept for elementary children from the 4^th^ to the 6^th^ grade in Taiwan. It has five subscales including Family, School, Appearance, Physical, and Emotion domains [11]. The internal consistency coefficients were reported to be .83 to .89 for the ESCS scores. Its test-retest reliability coefficients were reported to be .76 to .91 for the scores of its domains. The results of exploratory and confirmatory factor analyses were reported to support its hierarchal and multidimensional traits. Low to moderate correlations were found with sociometric test scores [42] as well as the Children’s Anxiety Scale scores [43].

#### The Behavior and Emotional Rating Scale (BERS)

The BERS is a 52-item scale for a child’s parents or teachers to fill out. Its purpose is to assess one’s emotional and behavioral strengths in five domains: Interpersonal Strengths, Family Involvement, Intrapersonal Strengths, School Functioning, and Affective Strengths [44]. Its Chinese version was translated by Yang [26]. The internal consistency estimates for the Chinese BERS scores were over .80, and the test-retest reliability coefficients were found as .73 to .88 for its five subscales. Strong to moderate correlations were found with the scores of the Self-perception Profile for Children [10], the Waller-McConnell Scale of Social Competence and School Adjustment [45], and the Child Behavior Checklist-Teacher Report Form [46].

### Analyses

All of the analyses were performed using SAS 9.3, except for the multi-group confirmatory factor analysis (MGCFA). Amos 19 was used for MGCFA. The internal consistency coefficients of the multimedia Piers-Harris 2 scores were calculated for evaluating score reliability. In order to assess convergent validity, correlations between the multimedia Piers-Harris 2 and the scores of the ESCS were calculated, two scales that both measure children’s self-concepts. Currently, there is no self-concept scale for children younger than 3^rd^ graders in Taiwan; therefore, a small subsample of 4^th^ graders was used for the convergence validity examination. Forty three 4^th^ graders were selected randomly to fill out the Piers-Harris 2 and the ESCS.

To investigate criterion validity, correlation between the scores of multimedia Piers-Harris 2 and the scores of the Behavior and Emotional Rating Scale (BERS) were calculated because previous research has demonstrated that self-concept may be related to behavioural/emotional variables [47–48]. Forty eight subjects across grades were selected, and their teachers filled out the BERS with regards to these children’s strengths relating to emotions and behavior. It was decided to use a small subsample, because it was difficult for teachers to fill out the BERS for the whole sample.

A MGCFA was conducted to evaluate the measurement invariance between the multimedia and paper-and-pencil versions of the 6-factor measurement model proposed by Piers and Herzberg [1]. Unweighted least squares (ULS) was used as estimation method because the items of Piers-Harris 2 are dichotomous [49]. Different levels of invariances between the two versions including configural, metric, and scale invariances as well as invariance of measurement errors were examined. Several model fit indices were calculated including the root mean square residual (RMR), standardized root mean square residual (SRMR), the global fit index (GFI), and the adjusted global fit index (AGFI). Hu and Bentler [50] suggested that a value of RMR or SRMR less than .08 and a value more than .9 for GFI and AGFI indicate an acceptable model fit. However, other researchers, like Browne and Cudeck, proposed that GFI and AGFI higher than .8 would also be acceptable [51]. Intraclass correlation coefficients (ICCs) were calculated to assess the equivalence between the multimedia and the paper-and-pencil versions, which is based on the model in which each scale is assessed by each rater, but the raters are the only raters of interest. Researchers also assessed the equality of the two versions' convergence and criterion-related validities. A statistical test for the difference between two independent corrections was used [52]. We used the one-tailed test (aα = .05) for all analyses except for the analyses in which the p value was adjusted for multiple tests.

## Results

### Psychometric Properties

#### Internal consistency coefficients

The Cronbach’s alphas for the scores of the multimedia Piers-Harris 2 are presented in Table 1. The alpha of the total score for the total sample is .91. The alphas of the total scores for the four grade strata range from .89 to .92, and the coefficient alphas for the scores of the six subscales range from .70 (POP) to .79 (BEH and FRE), respectively. For the scores of the six subscales throughout different grades, alphas range from .62 (POP for 2^nd^ grade) to .84 (FRE for 3^rd^ grade).

**Table 1:** Cronbach’s Alphas for the Scores of the Multimedia Piers-Harris 2.

	Cronbach’s Alphas				
	Total	Grade			
		1^st^	2^nd^	3^rd^	4^th^
Total	.91	.92	.89	.91	.91
Behavioral Adjustment	.79	.83	.66	.80	.81
Intellectual and School Status	.77	.80	.79	.76	.74
Physical Appearance and Attributes	.74	.70	.79	.79	.75
Freedom From Anxiety	.79	.76	.69	.84	.80
Popularity	.70	.69	.62	.71	.72
Happiness and Satisfaction	.77	.81	.67	.80	.78

*Note*. *N* = 248.

#### Interscale correlations

The results of the interscale correlations are shown in Table 2. The total scores demonstrate high correlations with the scores of the six domain scales, namely .80 (with BEH), .83 (with INT), .75 (with PHY), .82 (with FRE and POP both), and .77 (with HAP). The p value was adjusted for multiple tests using the Bonferroni correction. All scores in the six domain scales exhibit moderate correlations with each other (*rs* = .40 to .69).

**Table 2:** Interscale Correlations for the Multimedia Piers-Harris 2 Scores.

	1	2	3	4	5	6
Behavioral Adjustment	.80*	-				
Intellectual and School Status	.83*	.63*	-			
Physical Appearance and Attributes	.75*	.40*	.69*	-		
Freedom From Anxiety	.82*	.58*	.56*	.52*	-	
Popularity	.82*	.56*	.55*	.61*	.66*	-
Happiness and Satisfaction	.77*	.55*	.51*	.65*	.64*	.61*

*Note*. 1 = Total, 2 = Behavioral Adjustment, 3 = Intellectual and School Status, 4 = Physical Appearance and Attributes, 5 = Freedom from Anxiety, and 6 = Popularity.

*N* = 248

*p < .0025 (adjusted for multiple tests using the Bonferroni correction).

#### Convergent validity evidence

The results of the correlations between the multimedia Piers-Harris 2 scores and the ESCS scores are presented in Table 3. The total score of the multimedia Piers-Harris 2 shows a strong correlation with the total score of the ESCS (*r* = .76). The total score of multimedia Piers-Harris 2 is strongly correlated with the scores of the subscales of the ESCS except for the domain of family.

**Table 3:** Correlations between the Multimedia Piers-Harris 2 Scores and the Scores of the ESCS.

	Multimedia Piers-Harris 2						
	Total	Behavioral Adjustment	Intellectual and School Status	Physical Appearance and Attributes	Freedom from Anxiety	Popularity	Happiness and Satisfaction
ESCS							
Family	.40	.44	.41	.32	.27	.26	.29
School	.64*	.69*	.49*	.47*	.46	.54*	.40
Appearance	.59*	.58*	.39	.56*	.48*	.46	.44
Physical	.71*	.55*	.52*	.60*	.57*	.56*	.51*
Emotion	.68*	.56*	.43	.50*	.67*	.56*	.56*
Total	.76*	.70*	.57*	.62*	.61*	.60*	.55*

*Note*. *n* = 43.

*p < .001 (adjusted for multiple tests using the Bonferroni correction).

#### Correlation with BERS

The coefficients for the correlations of multimedia Piers-Harris 2 scores with the BERS scores are presented in Table 4. Before the p value was adjusted for the multiple tests, a moderate significance for the correlation between the total score of multimedia Piers-Harris 2 and the total score of the BERS as well as the correlations between the total score of multimedia Piers-Harris 2 and most of the subscale scores of the BERS were found. However, after the p value was adjusted for multiple tests, most of the significant relationships disappeared. Only the domains of behavioral adjustment and intellectual and school status are associated with some subscales of BERS.

**Table 4:** Correlations between the Multimedia Piers-Harris 2 Scores and the Scores of BERS.

	Multimedia Piers-Harris 2						
	Total	Behavioral Adjustment	Intellectual and School Status	Physical Appearance and Attributes	Freedom from Anxiety	Popularity	Happiness and Satisfaction
BERS							
Interpersonal	.23	.48*	.20	.02	.04	.18	.15
Family Involvement	.37	.39	.34	.33	.12	.29	.26
Intrapersonal	.44	.44	.47*	.39*	.10	.37	.36
School Functioning	.41	.48*	.46*	.28	.09	.35	.14
Affective	.31	.47*	.33	.17	.04	.23	.18
Total	.41	.47*	.45*	.31	.09	.33	.27

*Note*. *n* = 48.

*p < .001 (adjusted for multiple tests using the Bonferroni correction).

### Equivalence with the paper-and-pencil version

#### Multi-group Confirmatory factor analysis (MGCFA)

In terms of comparing the internal structure of the multimedia and paper-and-pencil versions of Piers-Harris 2 scores, a MGCFA was conducted. The model fit indices are listed in Table 5. Comparing the six-factor model between the multimedia and the paper-and-pencil versions of Piers-Harris 2, the results show that both the multimedia and the paper-and-pencil versions of Piers-Harris 2 have a clear and a distinct 6-factorial structure of children’s self-concept. According to Table 5, configural invariance is met (RMR = .01, SRMR = .08, GFI = .89, AGFI = .88) and the factor structures are therefore the same in the two groups. Metric invariance is reached, since the model fits coefficients are not deviated more than .01 compared with the configural invariance model. Regarding scalar invariance, invariance is met because the model fit coefficients are not deviated more than .01 compared with the metric invariance model [53], which indicates the factor loading and the intercepts (thresholds) are equal in both groups. Invariance of measurement errors also exists and the error variables of measurement models, factor covariances, and factor variances are identical across two groups.

**Table 5:** Results of Multiple Group Confirmatory Factor Analyses between the Multimedia and Paper-and-pencil Versions of Piers-Harris 2.

	RMR	SRMR	GFI	AGFI
Configural invariance	.01	.08	.89	.88
Metric invariance	.02	.09	.88	.87
Scale invariance	.02	.09	.87	.86
Invariance of measurement errors	.02	.09	.87	.86

*Note*. *N* = 248.

#### Intraclass correlation coefficients

Equivalence between the multimedia and paper-and-pencil forms of Piers-Harris 2 scores was also examined by calculating Intraclass Correlation Coefficients (ICCs) between the corresponding total score and the scores of the domain scales at two levels: the total study sample and grades (Table 6). The ICC for the total score of the total sample is .81. The ICCs for the four different grades range from .77 to .86. The ICCs for the scores of the domain scales of the total sample range from .65 to .78.

**Table 6:** Intraclass Correlation Coefficients (ICCs) for Equivalence between the Multimedia (MM) and Paper-and-pencil (PP) Piers-Harris 2 Scores.

	Equivalence MM-PP ICC				
	Total	Grade			
Scales	Sample	1^st^	2^nd^	3^rd^	4^th^
Total	.81	.77	.80	.86	.80
Behavioral Adjustment	.73	.68	.61	.79	.79
Intellectual and School Status	.78	.75	.80	.83	.71
Physical Appearance and Attributes	.73	.62	.73	.78	.78
Freedom From Anxiety	.74	.72	.59	.80	.74
Popularity	.65	.56	.68	.75	.60
Happiness and Satisfaction	.66	.65	.48	.70	.71

*Note*. *N* = 248.

#### The equality of convergence and criterion-related validities

The equality of convergence and criterion-related validities between the scores of the multimedia and paper-and-pencil versions of Piers-Harris 2 was assessed using a statistical test for the difference between two independent correlations [50]. There is no statistically significant difference between any of the correlations.

### Respondent Preference

The results of the survey for respondent preference show that more than half of the respondents (52%) preferred the multimedia version compared to the paper-and-pencil version (9%). Sixty three percent of the respondents think that the multimedia version is easier for them to answer in comparison to the paper-and-pencil version (9%). About three-fourths of the respondents (74%) are willing to answer the scale again with the computer-assisted version, but only one-third of them (35%) are willing to do so with the paper-and-pencil version.

## Discussion

The score reliability and validity evidence presented in this study suggests acceptable psychometric characteristics of the multimedia Piers-Harris 2 scores for this sample. According to Cicchetti’s standards [54], the internal consistency coefficient of the total score for the whole sample of .91 suggests an excellent level of score reliability. For the four grade strata, internal consistency coefficients of the total score for the first, third, and fourth grade were above .90, indicating an excellent level of score reliability. Internal consistency coefficient of the total score for the second grade was .89, suggesting that the level of score reliability was good. However, the high Cronbach alphas of the total scores could be a result of the high number of items.

The alphas were somewhat low for the scores of the subsample of 2^nd^ graders especially for the score of the Popularity domain scale. This result seemed to be related to the original item design of Piers-Harris 2. According to Piers and Herzberg [1], similar results were found for 2^nd^ graders in their study. It is possible that some items in some domain scales may be comprehended or interpreted differently by 2^nd^ graders. As discussed in the introduction, children aged 7 to 8 (2^nd^ graders) are in a developmental transition from early childhood to middle childhood. They are gradually developing their abilities to compare themselves with their peers [7]. This may have some effects on interpreting some items in regards to self-concept. Further investigation may be needed for improving the score stablility of the scores of a few domain scales such as Popularity for younger children.

The confirmatory factor analysis yielding six factors supported the multidimensional traits of the multimedia Piers-Harris 2 scores. This result also suggested that the factor structure, factor loadings, and intercepts underlying the Piers-Harris 2 were consistent across the multimedia and paper-and-pencil versions.

Our findings showed that the total score of the multimedia Piers-Harris 2 exhibited strong correlations with the scores of other domain scales, and all scores of the domain scales demonstrated moderate correlations with each other. These results suggest that these six subscales may reflect separate but also inter-correlated aspects of self-concept and provided evidence supporting the multidimensional and hierarchical characteristics of the multimedia Piers-Harris 2, which were consistent with prior studies [30, 55–57].

The total score of multimedia Piers-Harris 2 showed a strong correlation (*r* = .76) with the total score of the ESCS, which is satisfactory compared to prior similar studies (*r* = .34 to .73) [58–60]. This result supported the convergent validity of multimedia Piers-Harris 2. The total score of BERS is not significantly associated with the total score of multimedia Piers-Harris 2. Probably it is because BERS is not a good criterion to predict the scores of multimedia Piers-Harris. Future studies should assess the criterion validity of multimedia Piers-Harris 2 using other criterion [47–48, 61].

Sixty three (25.40% of the sample) first graders were recruited in this study, which seemed not to have affected the satisfactory results of reliability and validity examinations. The results of internal consistency of the total score and the scores of the six domain scales for the first grade (as shown in Table 1) also indicated its score stability. This study provided initial evidence supporting its applicability to younger children. However, further investigation should be conducted to determine its applicability to children younger than second grade.

The evidence supporting similarities of the internal structure of the multimedia and paper-and-pencil versions including the factor invariance, the ICCs, and the equality of convergence and criterion-related validities suggest that the scores of the multimedia and paper-and-pencil versions of Piers-Harris 2 seem to be similar. Regarding the factor invariance using MGCFA, the values of GFI and AGFI at all of the invariance levels were slightly lower, and the values of SRMR at the metric and scalar invariance levels and the values of SRMR for invariance of measurement errors were slightly higher (All were .09) according to the standards that Hu and Bentler proposed [50]. These three indices are known to be affected by the sample size [62]. Because the sample of this study is relatively small, it may have had some effects on these indices.

The results of ICCs show that the total score and the scores of the six domain scales for the entire sample ranged from good (> .60) to excellent (> .75) based on Cicchetti’s standards [54]. For the four grade strata, the ICCs for the total score were excellent (.77 to .86). Most of the ICCs for the scores of the six domain scales in the four different grades were at the level of good or excellent, except for three which were fair. These results suggested a high level of equivalence between the multimedia and paper-and-pencil formats of Piers-Harris 2 scores. However, many ICCs of the scores of the six domain scales for first and second grade showed lower values compared with the third and fourth grade. It is suggested to further investigate the possible influence of age difference on the score equivalence of these two formats. The results of assessing the equality of convergence and criterion-related validities suggested that the correlations were equivalent between the multimedia and paper-and-pencil versions of the Piers-Harris 2 scores. The overall results indicate acceptable psychometric qualities of the Chinese multimedia Piers-Harris 2 scores and provided further evidence of the applicability of the translated Piers-Harris 2 to Taiwanese children, consistent with previous studies mentioned earlier [27–28, 31–32].

It is not surprising that the majority of the children in this sample preferred completing the multimedia Piers-Harris 2. They also had higher motivation to retake it and viewed the computer version as easier. The results were similar with prior studies [63–65] investigating the preferences between the paper-and-pencil and computer formats. However, there were some limitations in this study. The present study only recruited 248 children from the three schools located in northern Taiwan. Therefore, generalization of its usefulness is limited. The design of a back-tracking function and a way of detecting unusual answering patterns or randomly-answering were missing. It may have slightly affected the reliability of the multimedia Piers-Harris 2. It should be considered to further investigate the possible factors influencing the score reliability level in different grade or age groups such as their developmental stages and language abilities.

In conclusion, this study provides several pieces of evidence in terms of the psychometric properties of the Chinese multimedia Piers-Harris 2 and suggests that the Chinese multimedia Piers-Harris 2 can be applied to Taiwanese children. The initial evidence also suggests that the scores of the paper-and-pencil and multimedia Piers-Harris 2 are equivalent. Children in this study prefer the multimedia Piers-Harris 2 over its conventional format, and a multimedia format can enhance children’s motivation of taking the Piers-Harris 2.
